# Silver linings: a qualitative study of desirable changes to cancer care during the COVID-19 pandemic

**DOI:** 10.3332/ecancer.2021.1202

**Published:** 2021-03-11

**Authors:** Dorothy Lombe, Richard Sullivan, Carlo Caduff, Zipporah Ali, Nirmala Bhoo-Pathy, Jim Cleary, Matt Jalink, Tomohiro Matsuda, Deborah Mukherji, Diana Sarfati, Verna Vanderpuye, Aasim Yusuf, Christopher Booth

**Affiliations:** 1 Cancer Diseases Hospital, Lusaka, 10101, Zambia; 2 Institute of Cancer Policy, King’s College London, London, WC2R 2LS, United Kingdom; 3 King’s College London, London, WC2R 2LS, United Kingdom; 4 Kenya Hospices and Palliative Care Association, Nairobi, 00202, Kenya; 5 Department of Social and Preventive Medicine, Faculty of Medicine, Universiti Malaya, Kuala Lumpur, 50603, Malaysia; 6 Department of Medicine and IU Simon Cancer Center, IU School of Medicine, Indianapolis, IN 46202, USA; 7 Division of Cancer Care and Epidemiology, Cancer Research Institute, Queen’s University, Kingston, K7L 3N6, Canada; 8 Population-based Cancer Registry Section, Division of Surveillance, Center for Cancer Control and Information Services, National Cancer Center, Tokyo, 104-0045, Japan; 9 Department of Internal Medicine, Division of Hematology-Oncology, American University of Beirut Medical Center (AUBMC), Beirut, 1107 2020, Lebanon; 10 Te Aho o Te Kahu, Cancer Control Agency, Wellington, 6011, New Zealand; 11 National Center for Oncology, Radiotherapy, and Nuclear Medicine, Accra, 00233, Ghana; 12 Shaukat Khanum Memorial Cancer Hospital and Research Centres, Lahore and Peshawar, 25100, Pakistan; a https://orcid.org/0000-0002-5083-1801

**Keywords:** silver linings, COVID-19, cancer care, barriers, policy change

## Abstract

**Introduction:**

Public health emergencies and crises such as the current COVID-19 pandemic can accelerate innovation and place renewed focus on the value of health interventions. Capturing important lessons learnt, both positive and negative, is vital. We aimed to document the perceived positive changes (silver linings) in cancer care that emerged during the COVID-19 pandemic and identify challenges that may limit their long-term adoption.

**Methods:**

This study employed a qualitative design. Semi-structured interviews (*n* = 20) were conducted with key opinion leaders from 14 countries. The participants were predominantly members of the International COVID-19 and Cancer Taskforce, who convened in March 2020 to address delivery of cancer care in the context of the pandemic. The Framework Method was employed to analyse the positive changes of the pandemic with corresponding challenges to their maintenance post-pandemic.

**Results:**

Ten themes of positive changes were identified which included: value in cancer care, digital communication, convenience, inclusivity and cooperation, decentralisation of cancer care, acceleration of policy change, human interactions, hygiene practices, health awareness and promotion and systems improvement. Impediments to the scale-up of these positive changes included resource disparities and variation in legal frameworks across regions. Barriers were largely attributed to behaviours and attitudes of stakeholders.

**Conclusion:**

The COVID-19 pandemic has led to important value-based innovations and changes for better cancer care across different health systems. The challenges to maintaining/implementing these changes vary by setting. Efforts are needed to implement improved elements of care that evolved during the pandemic.

## Introduction

The COVID-19 pandemic has caused massive global disruptions to health systems and economies. While this has been most pronounced in low resource settings, fragilities in high-income countries with strong public health care systems have also been exposed [1]. Health services for the diagnosis and management of cancer were not exempt from the impact of the first wave of the pandemic, despite the application of mitigation strategies by many cancer centres [2, 3]. The measures to combat transmission of severe acute respiratory syndrome coronavirus 2 (SARS-CoV-2) broadly grouped as non-pharmaceutical interventions (NPI) including major national lockdowns (‘stay at home orders’) have had significant impacts on delays to diagnosis and treatment of cancer for many patients [4]. The psycho-emotional aspects of cancer diagnosis demand a more personal interface between the healthcare providers as well as patient and physician relationships and the nature of the management regimens necessitate multiple hospital visits, all of which have been severely curtailed by NPI. Most national control efforts of the novel coronavirus have come at the expense of addressing wider health needs, particularly for cancer care.

Whilst most of the studies and literature to date have focused on the negative aspects of NPI on cancer care and outcomes, numerous positive examples (silver linings) have emerged. By these, we mean those pandemic- and lockdown-driven measures, which have improved cancer care. However, it must be noted that what constitutes a positive change in one setting may not necessarily be viewed in the same light in another setting. Also, the perception on the feasibility and benefits of these changes vary between different stakeholders and across socio-economic, cultural and technological environments. These underlying factors are ‘shadows’; i.e. measures that are wanted by stakeholders but are not feasible mainly due to resource and legal constraints.

In this study, we examined two paradigms of positive cancer care change during the COVID-19 pandemic. One is the decision-making to improve value-based care. The second is the maximal utilisation of basic and available resources to improve outcomes in all settings, particularly in low resource environments.

## Methods

This qualitative study involved semi-structured interviews with a diverse group of individuals working in cancer care across the globe. All but four of the participants were drawn from the International COVID-19 and Cancer Taskforce (covidcancertaskforce.org). The COVID-19 and Cancer Taskforce is a globally representative group of 30 cancer leaders spanning the disciplines of cancer across the care continuum (prevention, early detection, treatment and palliative care), and representatives of cancer centre networks and advocacy groups [5]. Working in a collaborative manner with expertise in infectious disease as well as non-communicable diseases, the taskforce seeks to assess the immediate impacts of the pandemic on cancer patients, services and healthcare workers as well as on treatment decision-making and patient/doctor interactions. It further addresses the risks for longer-term impacts on cancer outcomes, inequalities, research and affordability. Finally, the taskforce has tried to develop an integrated approach to build resilient cancer services able to respond to new pandemics, especially in low- and middle-income countries whose cancer services are particularly vulnerable to such unprecedented public health challenges [5].

The interviews were conducted in English over a video call (Zoom platform) secured with a passcode. A PowerPoint presentation with three directed questions and one general question guided the interviewee on the screen. Direct questions were: What were the positive changes perceived as a result of the pandemic? Should those changes be maintained after the pandemic? And what are the barriers to maintain the changes? The general question probed into any other related views held by the participant on the subject.

The perceived positive changes resulting from the pandemic were termed as silver linings. A pertinent question in the research was to understand the challenges that would preclude the maintenance of the silver linings. These challenges were divided into two fundamental groups termed as shadows and barriers. The term ‘shadows’ in this research represented factors that could not allow a silver linings change in one environment versus another due to systematic disparities. The term ‘barriers’ represent the challenges that could be overcome realistically with a directed intervention (Box 1).

The transcription of interviews was initially performed using secure online software [6]. Following this, it was proof read and corrected by a human transcriber. DL examined each participant’s transcript and audio recordings simultaneously to ensure the data was fully captured and this also presented an opportunity for data immersion. Data coding was then conducted by eliciting the keywords. Based on the framework analysis method, coding took an inductive-deductive approach as certain concepts were predefined from experience and conversations surrounding the pandemic [7, 8]. However, there were some unexpected data results that emerged and thus inductive coding was also employed making it a mixed approach.

The coded data were collated into an excel spreadsheet creating an analytical framework matrix. The rows were grouped into themes that were generated from the codes. The columns organised the concepts, the main one being the silver linings and two others representing shadows and barriers. Interviews continued until data saturation. Data saturation was reached when no new codes were elicited under the determined themes. Following saturation, indexing was applied to subsequent transcripts of the particular codes under the themes.

An iterative review of analytical framework was then performed to ensure no data had been ignored or omitted. The analytic framework matrix approach was appropriate as it linked silver linings to the shadows and barriers so as not to lose the realistic nature or feasibility in maintaining the changes. This identified crosschecking challenges forming the basis for contribution of the findings of this study to an accelerated recovery plan for cancer care from the shock of the prolonged COVID-19 pandemic.

The study was approved by the University of Zambia Biomedical Research Ethics Committee (REF.NO. 1065-2020) and the National Health Research Authority.

## Results

Twenty out of 25 cancer care professionals who were approached had accepted the invitation to participate in the study. The participants were from different disciplines including policy makers, epidemiologists, clinicians and medical physicists.

The representation of the participants by WHO regions and World Bank Income groups is presented in Table 1.

### Silver linings

Ten silver lining themes emerged including: value in cancer care, digital communication, convenience, inclusivity and cooperation, decentralisation of cancer care, acceleration of policy change, human interactions, hygiene practices, health awareness and promotion and systems improvement (Table 2).

Box 1. **Terminology.**

Silver linings – perceived positive changes in cancer care brought about by the COVID-19 pandemic

Shadows – Essential factors necessary to be present for the perceived positive changes brought about by COVID-19 pandemic that are not available in all environments despite the magnitude of benefit

Barriers – Essential factors that can be overcome realistically after identification regardless the resource setting.

The first interview question asked *‘What are the positive changes you have observed in cancer care as a result of the pandemic?’* Most participants reported that the COVID-19 had inadvertently increased value in cancer care.

Interviewee 1: ‘*Renewed interest in the concept of value in cancer care, in the idea that we really need to focus on giving treatments that make the biggest difference for the patients*.’

On the clinical front, due to lock-down restrictions and limited contact time, treatments with the largest magnitude of benefit were prioritised as physicians tried to balance the greater risk of mortality from COVID-19 versus cancer for the patient. An increase in honest conversations with patients for palliative care was noted. In some settings, there was a shifting of procedures to day surgeries, which maximised the use of resources and was cost saving both for the patient and the health system. In a bid to minimise hospital visits and patient contacts, chemotherapy delivery was simplified through the establishment of home-based chemotherapy and use of more convenient chemotherapy protocols (i.e. less frequent, oral agents).

In line with value, digital communication overlapped with convenience for patients, physicians, students, lecturers and researchers. Follow-ups have become more efficient, given the central location of most cancer centres, centres that employed telephone or web-based reviews allowed patients to avoid travel and wait times. For physicians, the greater flexibility in conducting telephonic reviews was suggested as an added benefit.

Researchers, students and lecturers appreciated the inclusivity and convenience associated with greater use of digital communication. Participants, particularly from lower and middle income countries (LMICs) and upper middle income countries (UMICs) acknowledged the wider access to academic and scientific meetings both locally and internationally due to a shift to virtual meetings. Not needing to obtain visas and pay for airfares was also acknowledged as a major benefit. The shift to digital communication presented an opportunity to learn new IT skills and transfer of knowledge.

Interviewee 2: ‘*I’m learning all these new skills. How to do Zoom and Microsoft Teams for online platforms.’*

‘So we’ve now been very effectively using things like voice over PowerPoint and getting used to things like online forum discussions, which is something that we’ve never done before but it has actually been really well received by the students especially as they are millennials.’ ‘Our students are all of millennial generation that are very, you know up to date with technology and very used to using technology and in some ways feel more confident to interact in a virtual space than they might do in a face-to-face lecture situations.’

*‘So I think this is a positive thing because it’s something that the older generation perhaps have never appreciated before that it actually improves the educational experience rather than being detrimental to that and in terms of the academic meetings*.’

The decreased need for work-related travel was favourably viewed especially for those with familial obligations at home (this was most commonly reported by women).

The acceleration of policy change was attributed to the pandemic for long outstanding issues such as decentralisation of cancer care, more convenient screening methods (e.g. HPV self-collection, stool for colon cancer screening) and out-dated limiting legal framework (home-based chemotherapy). The exposure of gaps in data collection has initiated rapid revisions in systems.

Participants also noted an increase in interaction and collaboration with ‘unlikely’ partners, e.g. the International COVID-19 and Cancer Taskforce and other modelling consortia that have emanated as a result of the pandemic. Increased awareness for health and the environment, employment of more stringent hygiene practices (hand washing, adherence to cleaning schedules, surface and machine wipe down) and systems improvements (attention to waiting times/appointments, throughput of patients) contributed to perceived low cost but extremely positive pandemic changes.

### Shadows

The second question asked *‘Do you think the changes should be maintained after the pandemic?’* This question evoked the reality of conditions surrounding the perceived positive changes, defining disparities rather than overt barriers (Table 3). For instance, the inability of radiation oncologists to shorten radiotherapy regimen to larger fraction per day due to poor quality machines with no multi-leaf collimators or a lack of cerrobend for shielding. The cheaper older machines also lack on-board imaging to make delivery of such large doses per fraction safe.

Legal frameworks in certain regions can pose bottlenecks outside emergency situations. Examples are laws restricting doctors from utilising digital communication for highly sensitive data, reimbursement for digital consultations or administering chemotherapy in patients’ homes.

Digital communication can also increase disparities, as not all stakeholders have access to the technology or the home environment is not suitable for virtual meetings and learning. Emotions and connection may also be missed as a result of a virtual interface.

Interviewee 16: *‘We know we can’t judge affect very well when we’re doing this. We can’t read people because we’re social animals and we need human beings in front of us to read effects.’*

Other cautions expressed in maintaining the pandemic status quo were: the effects of overuse of sanitisers are still unknown, increased exposure to chemicals in cleaning materials and its effect on the environment as well as burn on from being constantly engaged on the virtual platform.

### Barriers

The third question asked ‘What do you think are the barriers to maintain the changes?’ Barriers were interpreted as the surmountable changes that can be addressed by definitive steps once recognised (Table 4).

The most common barrier sited was behaviours and attitudes. Prescriptions of less fractions of radiotherapy or abstaining from prescribing systemic therapy of low value (expensive drugs with marginal clinical benefit) are economic behaviours that are dependent on providers.

Behavioural preferences for face-to-face interaction from patients, students and physicians can present a barrier as soon as restrictions are lifted. Some students have failed to appreciate the virtual platform of learning. Embracing new technology in clinical practice is not only a contention for older generation but can be noted across the board.

The justification for travel during conferences and opportunity for ‘time away’ and tourism was also mentioned. Invasion into personal space was also an issue raised for international meeting with invitees from different time zones.

Interviewee 16: *‘There are changes which ought to stay on; the question [of] whether they will or not I think is very much dependent on behaviors, and what I mean by behaviors is I mean just habits that clinical communities have grown into versus whether you’re allowed to do it or not.’*

## Discussion

Multiple publications have described the impact of the COVID-19 pandemic on cancer care, which largely focused on the challenges experienced. These reports are frequently regionalised with homogenous factors and conditions [9–11]. To the authors’ knowledge, the positive changes and innovations in cancer care have not been widely reported or studied across heterogeneous environments to assess their relevance and sustainability past the COVID-19 pandemic. This qualitative study sought to describe what lessons can be gained bi-directionally between different health systems during the pandemic. Cessation of low-value practices in cancer care has long been sought and the pandemic provided a window for implementation of the recommendations made by various groups [12–15].

The pandemic has led to enhanced use of digital technology. Continued interaction or provision of services relies on the different forms such as telephone or web-based. The benefits of its use has been noted both on the clinical and non-clinical front in cancer care. Guido-Estrada and Crawford [16] sited the benefits of telemedicine as convenience, satisfaction and cost saving, and providing an increase in physician autonomy and relaxation of regulatory restrictions on reimbursements [16]. The limitations that were echoed in the results of our study were physical examination, staff support and scheduling issues to allow for flexibility as well as disparities in availability of infrastructure necessary for all patients and physicians to participate in telemedicine.

Cancer care must be taken in context of the whole continuum of care including policy, which frames the building block of prevention, treatment, palliative care, research and training. Inevitably, research and training were expected to take a back seat in times of crisis to prioritise activities with high impact on morbidity and mortality but this would be short sighted in the context of a prolonged pandemic as we have experienced with COVID-19. A pragmatic outlook on continuing to build the body of knowledge and clinical skill through remote learning is imperative and possible [17, 18].

The crisis has also led to enhanced global cooperation, not only at the high-level political front, but from the scientific and civil worlds with the goal of fighting against a common enemy. The establishment of the International Taskforce for COVID-19 and Cancer brought experts from all regions of the world to draw from their diverse experience showcasing resilience and leadership. Using such cooperation, it is possible to share the best practices emanating in cancer care from the COVID-19 pandemic and investigate their application in accelerating the post pandemic recovery.

This study has characterised the diverse aspects of the perceived positive changes emanating in a time of COVID-19 and identified possible bottlenecks to their maintenance and scale up. The recommendations arising from the knowledge gained in this study to overcome the challenges in maintaining positive changes are approached from two directions. Firstly, for the shadows, the solutions are based on larger scale capacity building and infrastructure development. Whereas, the main recommendation to overcome the barriers is investment in behaviour and attitudes, for example, hygiene practices, prescription of high value clinical interventions and health systems practices, which are low cost and feasible.

This study has limitations. The participants were drawn from a group of experts at leadership level and as such the perspectives may not be reflective of all frontline cancer care professionals. The extent to which the identified themes are generalisable will be evaluated in a planned follow-up study which will use findings from the current study to undertake a broad global electronic survey. Another limitation was that the different regions were undergoing different levels of virus burden and therefore the local health system responses could not be accounted for. These limitations will be addressed in planned subsequent work of a survey to quantify the extent to which these results may be applicable across more diverse health systems.

## Conclusions

The COVID-19 pandemic has had a devastating impact on the world’s health systems directly impacting cancer care. However, the positive innovations and lessons learnt in this crisis must not dissipate post-COVID but must be systemically documented and maintained. The associated challenges (shadows and barriers) to maintain these positive changes must be redressed and integrated into global policy going forward.

## Conflicts of interest

There are no conflicts of interest to declare in this manuscript.

## Funding statement

This study was supported through the UK Research and Innovation GCRF RESEARCH FOR HEALTH IN CONFLICT (R4HC-MENA); developing capability, partnerships and research in the Middle and Near East (MENA) ES/P010962/1.

## Figures and Tables

**Table 1.** Characteristics of the interviewed cohort.

Speciality	Gender	Country	WHO region	World Bank group by income
Medical oncologist	Male	Canada	PAHO	High income
Medical oncologist	Female	Lebanon	EMRO	Upper middle income
Chief medical officer	Male	Pakistan	EMRO	Lower middle income
Public health medicine specialist	Female	Malaysia	WPRO	Upper middle income
Gynaecologic oncologist	Male	Zambia	AFRO	Lower middle income
Palliative care physician	Female	Kenya	AFRO	Lower middle income
Epidemiologist	Female	UK	EURO	High income
Clinical oncologist	Male	UK	EURO	High income
Director of cancer centre	Male	Columbia	PAHO	Upper middle income
Paediatric oncologist	Male	Turkey	EURO	Upper middle income
Epidemiologist	Female	Australia	WPRO	High income
Director of cancer centre	Male	India	SEARO	Lower middle income
Epidemiologist/statistician	Male	Japan	WPRO	High income
Clinical oncologist	Female	Ghana	AFRO	Lower middle income
National director, cancer control	Female	New Zealand	WPRO	High income
Director of cancer institute	Male	UK	EURO	High income
Haematologist	Male	Malaysia	WPRO	Upper middle income
Clinical oncologist	Female	Zambia	AFRO	Lower middle income
CEO palliative care	Male	Malaysia	WPRO	Upper middle income
Medical physicist	Female	Zambia	AFRO	Lower middle income

PAHO, Pan American Health Organisation; EMRO, Regional Office for the Eastern Mediterranean; WPRO, Regional Office for the Western Pacific; AFRO, Regional Office for Africa; EURO, Regional Office for Europe; SEARO, Regional Office for South-East Asia

**Table 2.** Silver linings themes and examples.

	Themes of silver linings	Examples of silver linings
1	Value in cancer care	Treatments that have the greatest magnitude of benefit
		Honest conversations – psychological aspects of palliative care
2	Digital communication (telephone/web-based)	Less travel for unnecessary visits and decongested clinics
		For collaborative effort amongst clinical researchers and multidisciplinary teams and access to meetings
3	Convenience	International collaboration easy – no need for visas, expensive travel costs
		For individuals with families who could not participate because it meant time away from home
4	Inclusivity and cooperation	NGOs and palliative care have gained prominence for role in community acting as a bridge between community and tertiary centres
		Team work across professional cadres (e.g. epidemiologists being valued in clinical scenario)
5	Decentralisation of cancer care	Shift to general practitioner/nurse led follow-up visits and imaging
		Community engagement in palliative care (which is the best model that has taken time to take root)
		Innovation in medicine distribution, home administration of chemotherapy
6	Policy change	Basis for change in legal framework prohibiting community based chemotherapy administration and use of digital technology in health
		Shift to more patient centred screening practices HPV – self-collection; stool-based testing for colon cancer
7	Human interactions	Easier consolidation of global collaborations and meeting of unlikely groups
		Collegial interactions are qualitative than quantitative because of limited time with increased communication efficiency
8	Hygiene practices	More hand washing, adherence to cleaning schedules
9	Health awareness and promotion	Increased awareness of anti-smoking messaging
		Increased exposure of fake news and false medical propaganda; increased trust in health experts
10	Systems improvement	Increased attention to waiting times/appointments
		Increased attention to patient throughput at different levels of service in hospitals

**Table 3.** Shadows to silver linings.

	Themes of silver linings	Examples of ‘shadows’ to the silver linings
1	Value in cancer care	Bigger fraction of radiotherapy require machines with high precision
		Legal framework
2	Digital communication (telephone/web-based)	Disparities in access – not all professionals and students have access to smart phone, reliable internet or the skills
		Misses some emotions and expressions that could change the care pattern
		Being connected 24 hours to meetings in different time zones takes away family time and there is usually no time off work allocated to virtual meetings
		Legal frame work
3	Convenience	Home environment not convenient for some
4	Inclusivity and cooperation	Unintended exposures to risks
5	Decentralisation of cancer care	Lack of human resource and infrastructure
6	Policy change	Established systems are harder to change as a small change in one place may have an unintended domino/collateral effect
7	Human interactions	Burn-out from increased expectation or responsibilities
8	Hygiene practices	Effects of overuse of sanitisers, increased exposure to chemicals in cleaning materials unknown
9	Health awareness and promotion	Some contradiction on initial data and expert opinions
10	Systems improvement	Costs of infrastructure and human resource

**Table 4.** Barriers to silver linings.

	Themes	Barriers to continuation after pandemic
1	Value in cancer care	Economic behaviour – Reimbursement for longer fractionation
2	Digital communication (telephone/web-based)	Behavioural preference from both patient and physicians for physical contact
		Students may feel not value for money through web based teaching
3	Convenience	Loss of team spirit as people lose physical workspace due to social distancing rules
4	Inclusivity and cooperation	Cultural barriers
5	Decentralisation of cancer care	Belief that best treatment is at tertiary level
6	Policy change	Policy makers may not be able to understand what happens on the ground due to lack of eloquent justification from technocrats
7	Human interactions	Eventually, the pandemic will be over and online meetings will revert to physical meetings with associated costs
8	Hygiene practices	Behavioural attitudes and relaxation
9	Health awareness and promotion	Lack of will power to break unhealthy habits; Distrust in mass information
10	Systems improvement	Behavioural attitudes
